# Plasma extracellular superoxide dismutase concentration, allelic variations in the *SOD3* gene and risk of myocardial infarction and all-cause mortality in people with type 1 and type 2 diabetes

**DOI:** 10.1186/s12933-014-0163-2

**Published:** 2015-01-15

**Authors:** Kamel Mohammedi, Naïma Bellili-Muñoz, Stefan L Marklund, Fathi Driss, Hervé Le Nagard, Thiago A Patente, Frédéric Fumeron, Ronan Roussel, Samy Hadjadj, Michel Marre, Gilberto Velho

**Affiliations:** INSERM, UMR_S 1138, Centre de Recherche des Cordeliers, 15 rue de l’École de Médecine, 75006 Paris, France; Assistance Publique Hôpitaux de Paris, DHU FIRE, Department of Diabetology, Endocrinology and Nutrition, Bichat Hospital, 46 rue Henri Huchard, 75018 Paris, France; Department of Medical Biosciences, Umeå University, Building 6M, 90185 Umeå, Sweden; INSERM, Research Unit 773, 16 rue Henri Huchard, 75018 Paris, France; Assistance Publique Hôpitaux de Paris, Department of Biochemistry, Bichat Hospital, 46 rue Henri Huchard, 75018 Paris, France; INSERM, Research Unit 1137 - IAME, 16 rue Henri Huchard, 75018 Paris, France; Laboratório de Endocrinologia Celular e Molecular (LIM-25), Faculdade de Medicina da Universidade de São Paulo (FMUSP), Avenida Dr. Arnaldo 455, CEP 01246903 São Paulo, SP Brazil; Sorbonne Paris Cité, UFR de Médecine, Université Paris Diderot, 16 rue Henri Huchard, 75018 Paris, France; Department of Endocrinology and Diabetology, Centre Hospitalier Universitaire de Poitiers, 2 Rue de la Milétrie, 86021 Poitiers, France; INSERM, Research Unit 1082, 2 Rue de la Milétrie, 86021 Poitiers, France; INSERM, CIC 1402, 2 Rue de la Milétrie, 86021 Poitiers, France; UFR de Médecine et Pharmacie, Université de Poitiers, 6 Rue de la Milétrie, 86073 Poitiers, France

**Keywords:** Oxidative Stress, SOD3, Myocardial Infarction, Mortality, Diabetes Mellitus

## Abstract

**Background:**

Oxidative stress is involved in development of diabetes complications. Extracellular superoxide dismutase (EC-SOD, SOD3) is a major extracellular antioxidant enzyme and is highly expressed in arterial walls. Advanced oxidation protein products (AOPP) and 8-iso-prostaglandin (isoprostane) are markers of oxidative stress. We investigated association of *SOD3* gene variants, plasma concentrations of EC-SOD, AOPP and isoprostane with myocardial infarction and mortality in diabetic patients.

**Methods:**

We studied three cohorts designed to evaluate the vascular complications of diabetes: the GENEDIAB study (469 participants with type 1 diabetes at baseline; follow-up data for 259 participants), the GENESIS study (603 participants with type 1 diabetes at baseline; follow-up data for 525 participants) and the DIABHYCAR study (3137 participants with type 2 diabetes at baseline and follow-up). Duration of follow-up was 9, 5, and 5 years, respectively. Main outcome measures were incidence of myocardial infarction, and cardiovascular and total mortality during follow-up. Six single nucleotide polymorphisms in the *SOD3* locus were genotyped in the three cohorts. Plasma concentrations of EC-SOD, AOPP, and isoprostane were measured in baseline samples of GENEDIAB participants.

**Results:**

In GENEDIAB/GENESIS pooled cohorts, the minor T-allele of rs2284659 variant was inversely associated with the prevalence at baseline (Odds Ratio 0.48, 95% CI 0.29–0.78, p = 0.004) and the incidence during follow-up of myocardial infarction (Hazard Ratio 0.58, 95% CI 0.40–0.83, p = 0.003) and with cardiovascular (HR 0.33, 95% CI 0.08–0.74, p = 0.004) and all-cause mortality (HR 0.44, 95% CI 0.21–0.73, p = 0.0006). The protective allele was associated with higher plasma EC-SOD and lower plasma AOPP concentrations in GENEDIAB. It was also inversely associated with incidence of myocardial infarction (HR 0.75, 95% CI 0.59–0.94, p = 0.01) and all-cause mortality (HR 0.87, 95% CI 0.79–0.97, p = 0.008) in DIABHYCAR.

**Conclusions:**

The T-allele of rs2284659 in the promoter of *SOD3* was associated with a more favorable plasma redox status and with better cardiovascular outcomes in diabetic patients. Our results suggest that EC-SOD plays an important role in the mechanisms of vascular protection against diabetes-related oxidative stress.

**Electronic supplementary material:**

The online version of this article (doi:10.1186/s12933-014-0163-2) contains supplementary material, which is available to authorized users.

## Introduction

Diabetes mellitus is associated with increased mortality rates [1,2]. Despite significant improvement of medical care during late decades, life expectancy of patients with type 1 or type 2 diabetes remains reduced as compared to age- and sex-matched nondiabetic subjects [1,2]. Cardiovascular disease is the leading cause of mortality and morbidity in patients with diabetes [1,2], and diabetic patients have a 3-fold higher risk than nondiabetic individuals of developing atherosclerosis and its clinical complications [1,3]. It is now well established that susceptibility to cardiovascular complications in diabetic patients are modulated by genetic factors [4].

Oxidative stress plays a major role in the development of microvascular and macrovascular complications of diabetes [5]. Superoxide dismutase (SOD) is a class of enzymes that catalyze the dismutation of superoxide into oxygen and hydrogen peroxide [6], and modulate the redox status of eukaryotic cells. The extracellular superoxide dismutase (EC-SOD or SOD3) is a major extracellular antioxidant enzyme, mainly located in the lymph, synovial fluid and plasma [7]. EC-SOD is highly expressed in blood vessels, particularly in arterial walls and represents up to 70% of the SOD activity in this tissue [7]. EC-SOD plays an important role in the protection against cardiovascular oxidative stress [8,9]. Advanced oxidation protein products (AOPP) and 8-iso-prostaglandin (isoprostane) were identified as markers of oxidative stress in patients with heart disease [10,11]. In the present study we investigated associations of allelic variation in the *SOD3* gene with the risk of myocardial infarction, cardiovascular death and all-cause mortality in two prospective cohorts of type 1 diabetic patients and one prospective cohort of type 2 diabetic patients. Correlations of genotypes, clinical outcomes, and circulating levels of EC-SOD, AOPP and isoprostane were also studied in type 1 diabetic participants.

## Methods

### Participants: GENEDIAB and GENESIS cohorts of type 1 diabetic patients

We studied two multicenter binational (Belgium and France) cohorts of type 1 diabetes patients designed to study the vascular complications of diabetes. The GENEDIAB (*GEnétique de la NEphropathie DIABétique*) study was conducted in 494 people with type 1 diabetes and pre-proliferative or proliferative retinopathy [12]. GENESIS France-Belgium was a family-based study including 662 probands with type 1 diabetes and non-proliferative or proliferative retinopathy, and 578 first-degree relatives [13]. In the present investigation, we analyzed at baseline 469 GENEDIAB participants and 603 GENESIS probands for whom DNA samples were available. Clinical characteristics of GENEDIAB and GENESIS participants are shown in the Additional file 1: Table S1. In a prospective observational study, subsets of GENEDIAB (n = 261) and GENESIS (n = 549) participants were followed until an end point was reached, or until February 2007. The subsets were composed of participants who attended outpatient clinics at least once during the follow-up period. In the present investigation, we analyzed follow-up data from 259 GENEDIAB participants and 525 GENESIS probands for whom DNA samples were available. Mean duration of follow-up was 9 ± 3 and 5 ± 2 years, respectively.

### Participants: the DIABHYCAR cohort of type 2 diabetic patients

DIABHYCAR *(non-insulin-dependent DIABetes, HYpertension, microalbuminuria or proteinuria, CArdiovascular events, and Ramipril)* was a multinational clinical trial conducted in patients with type 2 diabetes selected on the basis of persistent microalbuminuria (urinary albumin excretion, UAE = 20-200 mg/l) or macroalbuminuria (UAE > 200 mg/l) without renal failure (plasma creatinine <150 μmol/l) at baseline [14]. The trial tested whether a low dose of ramipril, an angiotensin converting enzyme (ACE) inhibitor, able to reduce UAE would also reduce cardiovascular and/or renal events such as myocardial infarction, stroke, acute heart failure, end-stage renal disease (ESRD), and cardiovascular death. The median duration of follow-up was 4.7 years. Results were negative regarding the drug effect and were published previously [14]. In the present work, we studied 3137 French participants of DIABHYCAR. Study protocols of the three cohorts were approved by the ethics committee of the University Hospital of Angers, France. All participants gave written informed consent.

### Outcomes

Myocardial infarction was diagnosed as the occurrence of at least 2 out of 3 of the following criteria: constrictive chest pain lasting 20 minutes or longer, increased serum creatinine phosphokinase and/or troponine levels, or typical electrocardiographic changes. All-cause mortality was defined as death from any cause occurring during follow-up. Cardiovascular death was defined as sudden death, death following myocardial infarction, congestive heart failure and arrhythmias.

### Clinical and laboratory procedures

Estimation of the glomerular filtration rate (eGFR) was computed with the Modification of Diet in Renal Disease (MDRD) formula. Plasma concentrations of EC-SOD, AOPP and isoprostane were measured in 440 GENEDIAB participants for whom fasting plasma-EDTA samples were available. Samples were collected at baseline and kept frozen at −80°C. EC-SOD was measured by ELISA as described previously [15]. Isoprostane was measured by ELISA with a commercial kit (15-Isoprostane F2t ELISA Kit EA84, Oxford Biomedical Research). AOPP were measured by spectrophotometry using a microplate reader (MR 5000, Dynatech, Paris, France) [16]. Results were expressed as ng/ml (EC-SOD and isoprostane) and μmol/L of chloramine-T equivalents (AOPP).

Five single nucleotide polymorphism (SNP) in the *SOD3* gene region (chromosome 4q21) were chosen in HapMap (public release #23) on the basis of giving information on ~90% of the allelic variation of SNPs with minor allele frequency ≥5% at r^2^ > 0.8 in haplotype blocks containing *SOD3*: rs2284659 (promoter, ~2.3 kb 5’ from exon 1 start), rs2695234, rs17552548, rs758946 and rs2270224 (respectively ~1.8, ~2.3, ~4.8 and ~5.9 kb 3’ from the end of exon 2/UTR). The rare missense functional variant rs1799895 (Arg213Gly, exon 2) was also genotyped [17]. Genotypes were determined by competitive allele-specific PCR genotyping system assays (KASP, LGC Genomics, Hoddeston, UK). Genotyping success rate was >92%. Genotyping was repeated in 5% of subjects with 100% concordance. Genotypes were in Hardy-Weinberg equilibrium (Pearson’s chi-squared test with 1 degree of freedom p > 0.05) except for rs2695234 and rs2270224 in the DIABHYCAR cohort.

### Statistical analysis

Results are expressed as mean ± SEM except where stated otherwise. Differences between groups were assessed by analysis of variance (ANOVA), analysis of covariance (ANCOVA), and contingency table chi-square test. Genetic associations were assessed by regression models. Cox proportional hazards survival regression analyses were used to examine the effect of explanatory variables on time-related survival (or myocardial infarction-free) rates in prospective analyses. Logistic regression analyses were used for cross-sectional analyses. Hazard ratios (HR) or odds ratios (OR), respectively, with their 95% confidence intervals (CI) were computed for the minor alleles. The choice of a genetic model (dominant, codominant or recessive) for each of the analyses was based on the prevalence and/or incidence of the traits (myocardial infarction, all-cause mortality) by genotype. HR or OR were not computed for SNP with minor allele frequency (MAF) lower than 2% in the groups being compared, nor for genotypes not in Hardy-Weinberg equilibrium. Data were log-transformed for the analyses when the normality of the distribution was rejected by the Shapiro-Wilk W test. To increase statistical power, genetic analyses in type 1 diabetic participants were performed in GENEDIAB and GENESIS pooled studies with appropriate covariate adjustments to take into account cohort differences. Correction for multiple comparisons due to multiple SNP testing took into account the effective number of independent tests (Meff) based on the degree of linkage disequilibrium between SNPs [18]. Thus, p ≤ 0.01 was considered significant, unless stated otherwise. The power to detect allelic associations with the prevalence and the incidence of myocardial infarction, and with all-cause mortality in the GENEDIAB/GENESIS pooled study, was 90, 82 and 81%, respectively, for hazard ratio ≥1.5 and alpha = 0.01. It was 99% for each outcome in DIABHYCAR cohort. Statistics were performed with the JMP software (SAS Institute Inc., Cary, NC).

## Results

### GENEDIAB and GENESIS cohorts: previous myocardial infarction at baseline by *SOD3* genotype

The prevalence of previous myocardial infarction at baseline in the pooled cohorts of patients with type 1 diabetes was 5.8% (n = 62). Genotype frequencies by history of previous myocardial infarction at baseline are shown in the Additional file 1: Table S2. The prevalence of myocardial infarction by rs2284659 genotype was 7.0% (GG), 5.4% (GT) and 2.2% (TT) suggesting a protective effect of the minor T-allele in a codominant model. A logistic regression analysis confirmed the inverse association of the T-allele with the prevalence of previous myocardial infarction (OR 0.48, 95% CI 0.29–0.78, p = 0.004, in a codominant model adjusted for sex, age, duration of diabetes, use of antihypertensive and lipid lowering medications, and for cohort membership).

### GENEDIAB and GENESIS cohorts: clinical outcomes during follow-up

Characteristics of participants at baseline by the incidence of clinical outcomes during follow-up are shown in Table 1. Forty nine new cases of myocardial infarction occurred during follow-up. Its cumulative incidence was 6.3% (incidence rate 1.0 per 100 person-years). Individuals who had a myocardial infarction, compared to those who did not, were older, had a longer duration of diabetes, higher systolic blood pressure, lower eGFR, and were more likely to be of the male sex and to be taking antihypertensive and lipids lowering drugs. Previous myocardial infarction was more frequent at baseline in participants who developed new cases of myocardial infarction during the follow-up, as compared to those who did not. Death occurred in 72 participants during the follow-up. The cumulative incidence of total mortality was 9.2% (incidence rate 1.4 per 100 person-years). Causes of death included cardiovascular complications (38.9%), infectious diseases (15.3%), cancer (9.7%), acute metabolic complications (8.3%), kidney complications (2.8%) and other or undetermined etiologies (25.0%). Participants who died during follow-up, as compared to subjects who survived, were older, more frequently of the male sex, had a longer duration of diabetes, higher HbA1c, systolic and diastolic blood pressure, and UAE levels. They had lower eGFR and were more likely to have a previous history of tobacco smoking, hypertension and myocardial infarction.

**Table 1:** **GENEDIAB/GENESIS pooled study: Characteristics of participants at baseline by the incidence of myocardial infarction and all-cause mortality during follow-up**

	**Myocardial Infarction during follow-up**			**All-cause mortality**		
	**No**	**Yes**	**p**	**No**	**Yes**	**p**
N	735	49		712	72	
Male sex (%)	53.8	69.4	0.03	53.7	70.8	0.005
Age (years)	42.6 ± 11.0	52.3 ± 12.5	<0.0001	42.7 ± 11.1	49.5 ± 12.6	<0.0001
Duration of diabetes (years)	27.1 ± 9.0	33.3 ± 11.6	0.0004	27.2 ± 9.1	31.3 ± 10.6	0.004
Body mass index (kg/m^2^)	24.2 ± 3.5	24.9 ± 3.7	0.24	24.4 ± 3.5	23.6 ± 3.8	0.07
Systolic blood pressure (mmHg)	134 ± 19	144 ± 21	0.0007	134 ± 19	142 ± 18	0.0005
Diastolic blood pressure (mmHg)	77 ± 11	80 ± 11	0.07	76 ± 11	80 ± 10	0.004
HbA1c (%) and (mmol/mol)	8.5 ± 1.5 (70 ± 16)	8.9 ± 1.5 (74 ± 16)	0.08	8.5 ± 1.4 (69 ± 15)	9.4 ± 2.3 (79 ± 25)	<0.0001
Urinary albumin excretion (mg/l)	21 (177)	43 (836)	0.22	19 (157)	188 (900)	<0.0001
eGFR (ml/min)	86 ± 47	70 ± 31	0.02	86 ± 46	69 ± 56	<0.0001
Total cholesterol* (mmol/l)	5.67 ± 1.51	5.91 ± 1.07	0.28	5.65 ± 1.42	5.76 ± 1.67	0.97
Lipids lowering therapy (%)	7.9	16.3	0.04	8.3	11.1	0.41
Tobacco smoking (%)	42.0	47.9	0.42	41.2	59.2	0.004
Arterial hypertension (%)	60.8	83.7	0.001	60.5	84.7	<0.0001
Previous myocardial infarction (%)	3.8	22.9	<0.0001	3.9	21.1	<0.0001

Results expressed as mean ± SD, except urinary albumin excretion (UAE) expressed as median and interquartile range. Statistics of quantitative parameters are ANOVA performed with log-transformed data, or Wilcoxon test (UAE). eGFR: estimated glomerular filtration rate. *Data available only in the GENEDIAB cohort (n = 247). p < 0.05 is significant.

### GENEDIAB and GENESIS cohorts: clinical outcomes during follow-up by *SOD3* genotypes

Genotype frequencies by the incidence of clinical outcomes during follow-up are shown in Table 2. The incidence of myocardial infarction by rs2284659 genotype was 7.6% (GG), 5.4% (GT) and 5.6% (TT), suggesting a protective effect of the minor T-allele in a dominant model. Cox proportional hazards survival regression analysis confirmed the inverse associations of rs2284659 T-allele with the incidence of myocardial infarction (HR 0.58, 95% CI 0.40–0.83, p = 0.003, in a dominant model, adjusted for sex, age, duration of diabetes, blood pressure, HbA1c, UAE, eGFR, use of antihypertensive and lipid lowering medications, tobacco smoking and cohort membership). The incidence of all-cause mortality by rs2284659 genotype was 8.9% (GG), 10.3% (GT) and 4.3% (TT), suggesting a protective effect of the minor T-allele in a recessive model. The association was confirmed by a Cox proportional hazards survival regression analysis (HR 0.44, 95% CI 0.21–0.73, p = 0.0006, for the T-allele in a recessive model adjusted for the same covariates as above). The T-allele protective effect remained significant when we considered only the cases of cardiovascular death (HR 0.33, 95% CI 0.08–0.74, p = 0.004). No other significant allelic association was observed with any of the outcomes.

**Table 2:** **GENEDIAB/GENESIS pooled study: genotype frequency of** ***SOD3*** **variants by the incidence of myocardial infarction and all-cause mortality during follow-up**

	**Myocardial Infarction during follow-up**				**All-cause mortality**			
	**No**	**Yes**	**HR (95% C.I.)**	**p**	**No**	**Yes**	**HR (95% C.I.)**	**p**
rs2284659								
GG	0.413 (268)	0.500 (22)	0.45 (0.25 – 0.77)	0.003	0.412 (265)	0.413 (26)	0.44 (0.21 – 0.73)	0.0006*
GT	0.456 (297)	0.386 (17)			0.448 (289)	0.524 (33)		
TT	0.131 (85)	0.114 (5)			0.140 (90)	0.063 (4)		
MAF	0.359	0.307			0.364	0.325		
rs1799895								
CC	0.983 (638)	1.000 (42)	-	**	0.983 (628)	1.000 (65)	-	**
CG	0.017 (11)	0 (0)			0.017 (11)	0.000 (0)		
GG	0	0 (0)			0	0 (0)		
MAF	0.008	0			0.009	0		
rs2695234								
GG	0.861 (566)	0.930 (40)	0.54 (0.09 – 1.70)	0.34	0.858 (558)	0.922 (59)	0.65 (0.32 – 1.08)	0.10
GA	0.130 (85)	0.047 (2)			0.133 (86)	0.062 (4)		
AA	0.009 (6)	0.023 (1)			0.009 (6)	0.016 (1)		
MAF	0.074	0.047			0.076	0.047		
rs17552548								
AA	0.894 (575)	0975 (39)	-	**	0.904 (574)	0.857 (54)	1.47 (0.99 – 2.06)	0.06
GA	0.104 (67)	0.025 (1)			0.094 (60)	0.143 (9)		
GG	0.002 (1)	0 (0)			0.002 (1)	0 (0)		
MAF	0.054	0.013			0.049	0.071		
rs758946								
TT	0.857 (553)	0.930 (40)	0.49 (0.08 – 1.52)	0.25	0.853 (544)	0.936 (59)	0.68 (0.33 – 1.13)	0.15
TC	0.135 (87	0.047 (2)			0.138 (88)	0.064 (4)		
CC	0.008 (5)	0.023 (1)			0.009 (6)	0 (0)		
MAF	0.075	0.047			0.078	0.032		
rs2270224								
GG	0.270 (169)	0.136 (6)	1.12 (0.65 – 1.91)	0.68	0.257 (158)	0.303 (20)	0.75 (0.50 – 1.04)	0.09*
GA	0.483 (304)	0.682 (30)			0.495 (306)	0.530 (35)		
AA	0.247 (155)	0.182 (8)			0.248 (153)	0.167 (11)		
MAF	0.489	0.523			0.496	0.432		

Genotype data expressed as frequency and (number of cases). SNPs are sorted in 5’ to 3’ order. Hazards ratio for the minor allele in a dominant or recessive* model, determined in Cox proportional hazards survival regressive model, adjusted for sex, age, duration of diabetes, blood pressure, HbA1c, UAE, eGFR, use of antihypertensive and lipid lowering medications, tobacco smoking and cohort membership. p ≤ 0.01 is significant. MAF: minor allele frequency. **Statistics not computed due to low MAF (<0.02).

### GENEDIAB cohort: baseline correlations of EC-SOD and markers of oxidative stress

Plasma EC-SOD concentration at baseline was higher in women than in men (226 ± 18 vs 176 ± 11 ng/ml, p = 0.0004). It was positively correlated with systolic (R^2^ = 0.04, p < 0.0001) and diastolic blood pressure (R^2^ = 0.03, p < 0.0001), UAE (R^2^ = 0.03, p = 0.001), total cholesterol (R^2^ = 0.03, p = 0.0001), and inversely correlated with the body mass index (BMI; R^2^ = 0.02, p = 0.004) and eGFR (R^2^ = 0.12, p = 0.001). A stepwise regression analysis was performed to evaluate the independence of these correlations (Additional file 1: Table S3). Plasma EC-SOD concentration was entered in the model as the dependent variable. Age, duration of diabetes, HbA1c and the above mentioned variables were entered as independent covariates. Only eGFR, sex, systolic blood pressure, and BMI remained significantly correlated with EC-SOD and explained ~20% of the variance of the trait.

Plasma AOPP concentration at baseline was higher in women than in men (70 ± 3 vs 63 ± 2 μmol/l, p = 0.02), and was lower in subjects treated by ACE inhibitors (46 ± 3 vs 80 ± 2 μmol/l, p < 0.0001) or antihypertensive drugs (57 ± 36 vs 79 ± 29 μmol/l, p < 0.0001) than in subjects not receiving these medications. It was positively correlated with total cholesterol levels (R^2^ = 0.05, p < 0.0001). Only the use of ACE inhibitors, and total cholesterol levels remained significantly correlated with AOPP concentration in a stepwise regression analysis and explained 38% and 6%, respectively, of its variance (data not shown). Plasma isoprostane concentration was higher in subjects treated by lipid lowering drugs than in those who were not (2.13 ± 0.20 vs 1.61 ± 0.05 ng/ml, p = 0.005). It was positively correlated with diastolic blood pressure (R^2^ = 0.02, p = 0.004).

### GENEDIAB cohort: EC-SOD and markers of oxidative stress at baseline by clinical outcomes during follow-up

Circulating levels of EC-SOD, AOPP and isoprostane at baseline by clinical outcomes during follow-up are shown in Table 3. Plasma EC-SOD concentration was not significantly associated with clinical outcomes during follow-up. Plasma AOPP concentration was higher in patients who died during the follow-up as compared to those who survived. Plasma isoprostane concentration was significantly higher in subjects who presented a clinical outcome (myocardial infarction or death from any cause) during follow-up as compared to those who did not.

**Table 3:** **GENEDIAB cohort: Plasma EC-SOD, AOPP and Isoprostane concentrations by clinical outcomes during follow-up**

	**N**	**EC-SOD (ng/ml)**	**N**	**AOPP (μmol/l)**	**N**	**Isoprostane (ng/ml)**
Myocardial infarction						
Yes	25	194 ± 44	26	60 ± 6	26	2.37 ± 0.24
No	208	196 ± 15	214	66 ± 2	226	1.52 ± 0.08
p		0.51		0.49		0.0003
All-cause mortality						
Yes	47	195 ± 30	40	73 ± 6	42	2.22 ± 0.20
No	194	192 ± 14	209	56 ± 4	221	1.71 ± 0.12
p		0.57		0.004		0.002

Results expressed as mean ± SEM. Statistics are ANCOVA adjusted for sex, age, BMI, systolic blood pressure and eGFR (EC-SOD), for sex, age, total cholesterol concentration, and use of ACE inhibitors (AOPP), and for sex, age, diastolic blood pressure, and use of lipid lowering drugs (isoprostane). p < 0.05 is significant.

### GENEDIAB cohort: EC-SOD and markers of oxidative stress at baseline by *SOD3* genotypes

Genotype-related effects on EC-SOD, AOPP and isoprostane are shown in Table 4. Carriers of the protective T-allele of rs2284659 had significantly higher plasma EC-SOD concentration than homozygous GG carriers. Moreover, carriers of the TT genotype had significantly lower plasma AOPP concentration than G-allele carriers. Heterozygous carriers of the rare G-allele of the missense (Arg213Gly) functional variant rs1799895 had a ~6-fold increase in plasma EC-SOD concentration. Higher plasma AOPP concentration was also observed for GG carriers of rs2270224 and G-allele carriers of rs17552548 (p < 0.0001; data not shown). We observed no genotype-related association with plasma isoprostane concentration.

**Table 4:** **GENEDIAB cohort: Plasma EC-SOD, AOPP and Isoprostane concentrations at baseline by** ***SOD3*** **genotypes**

	**EC-SOD (ng/ml)**	**AOPP (μmol/l)**	**Isoprostane (ng/ml)**
rs2284659			
GG (n)	165 ± 17 (165)	66 ± 3 (156)	1.98 ± 0.11 (162)
GT (n)	230 ± 16 (172)	64 ± 3 (171)	1.92 ± 0.11 (181)
TT (n)	222 ± 25 (74)	55 ± 4 (73)	1.98 ± 0.16 (73)
p	0.007	0.02	0.90
rs1799895			
CC (n)	169 ± 6 (400)	64 ± 2 (390)	1.93 ± 0.10 (407)
CG (n)	1156 ± 32 (12)	52 ± 10 (12)	1.78 ± 0.34 (12)
p	<0.0001	0.34	0.41

Results expressed as mean ± SEM. Statistics are ANCOVA adjusted for sex, age, BMI, systolic blood pressure and eGFR (EC-SOD), for sex, age, total cholesterol concentration, and use of ACE inhibitors (AOPP), and for sex, age, diastolic blood pressure, and use of lipid lowering drugs (isoprostane). p ≤ 0.01 is significant.

### DIABHYCAR study: clinical outcomes during follow-up by *SOD3* genotype

Myocardial infarction was diagnosed in 95 participants and death occurred in 456 participants during the follow-up of the DIABHYCAR type 2 diabetes study. The cumulative incidence of myocardial infarction and total mortality was 3.0% and 14.5%, respectively, and their incidence rate was 0.7 and 3.3 per 100 person-years. Causes of death included cardiovascular complications and sudden death (40.0%), infectious diseases (2.4%), cancer (26.5%), acute metabolic complications (0.2%), end stage renal disease (ESRD, 0.9%) and other or undetermined etiologies (30.0%). Characteristics of participants at baseline by the incidence of myocardial infarction and all-cause mortality during the follow-up are shown in Table 5. Patients who died during follow-up, as compared to those who survived, were older, more frequently of the male sex, had a longer duration of diabetes, higher systolic blood pressure and UAE levels. They had lower BMI and eGFR levels, and were more likely to be taking antiplatelet drugs, and to have a previous history of hypertension and myocardial infarction. Genotype frequencies by the incidence of clinical outcomes during follow-up are shown in Additional file 1: Table S4. The incidence of outcomes by rs2284659 genotype was 3.6% (GG), 2.6% (GT) and 2.3% (TT) for myocardial infarction, and 16.3% (GG), 13.4% (GT) and 14.1% (TT) for all-cause mortality, suggesting a protective effect of the minor T-allele in a dominant model. Cox proportional hazards survival regression analyses confirmed the inverse associations of rs2284659 T-allele with the incidence of myocardial infarction (HR 0.75, 95% CI 0.59–0.94, p = 0.01) and all-cause mortality (HR 0.87, 95% CI 0.79–0.97, p = 0.008) in a dominant model, adjusted for sex, age, duration of diabetes, BMI, blood pressure, UAE, eGFR, hypertension and antiplatelet drugs and study treatment. A nominal inverse association was also observed when we considered only cardiovascular causes of death (HR 0.83, 95% CI 0.69–0.99, p = 0.03, for the T-allele in a dominant model, same adjustments as above).

**Table 5:** **DIABHYCAR cohort: Characteristics of participants at baseline by the incidence of myocardial infarction and all-cause mortality during follow-up**

	**Myocardial infarction during follow-up**			**All-cause mortality**		
	**No**	**Yes**	**p**	**No**	**Yes**	**p**
N	3042	95		2554	438	
Male sex (%)	73.0	75.8	0.54	72.2	77.2	0.03
Age (years)	65.6 ± 8.3	65.9 ± 8.8	0.79	64.8 ± 8.1	70.5 ± 8.5	<0.0001
Duration of diabetes (years)	10.4 ± 7.7	11.3 ± 8.6	0.49	10.1 ± 7.6	12.3 ± 8.2	<0.0001
Body mass index (kg/m2)	29.4 ± 4.6	29.1 ± 4.2	0.60	29.5 ± 4.6	28.8 ± 4.9	0.0008
Systolic blood pressure (mmHg)	144 ± 13	146 ± 16	0.14	144 ± 13	146 ± 13	0.007
Diastolic blood pressure (mmHg)	82 ± 8	81 ± 8	0.29	82 ± 8	82 ± 8	0.99
HbA1c (%) and (mmol/mol)	7.9 ± 1.8 (62 ± 19)	8.2 ± 1.8 (66 ± 20)	0.06	7.8 ± 1.7 (62 ± 19)	8.0 ± 2.0 (64 ± 22)	0.35
Urinary albumin excretion (mg/l)	76 (144)	78 (162)	0.92	72 (130)	109 (278)	<0.0001
eGFR (ml/min)	78 ± 22	75 ± 20	0.14	78 ± 22	72 ± 21	<0.0001
Total cholesterol (mmol/l)	5.79 ± 1.07	6.00 ± 1.25	0.18	5.78 ± 1.05	5.84 ± 1.15	0.37
HDL- cholesterol (mmol/l)	1.32 ± 0.36	1.25 ± 0.26	0.15	1.32 ± 0.35	1.29 ± 0.34	0.17
LDL- cholesterol (mmol/l)	3.52 ± 0.88	3.72 ± 1.02	0.10	3.53 ± 0.87	3.51 ± 0.92	0.61
Triglycerides (mmol/l)	1.83 (1.36)	1.81 (1.77)	0.53	1.84 (1.32)	1.84 (1.49)	0.69
Lipids lowering therapy (%)	35.5	33.7	0.71	36.0	32.2	0.12
Antiplatelet drugs (%)	18.8	16.8	0.63	17.0	28.5	<0.0001
Tobacco smoking (%)	14.4	13.7	0.84	14.2	14.6	0.83
Arterial hypertension (%)	56.1	60.0	0.44	54.9	64.4	0.0002
Previous myocardial infarction (%)	5.3	10.5	0.03	4.9	8.5	0.003

Results expressed as mean ± SD, except urinary albumin excretion (UAE) and triglycerides expressed as median and interquartile range. Statistics of quantitative parameters are ANOVA performed with log-transformed data, or Wilcoxon test (UAE, triglycerides). eGFR: estimated glomerular filtration rate. p < 0.05 is significant.

## Discussion

In the present investigation, we observed associations of a variant in the promoter region of the *SOD3* gene with cardiovascular morbidity and mortality in prospective cohorts of type 1 and type 2 diabetic patients. The minor T-allele of rs2284659 was inversely associated with the prevalence of myocardial infarction at baseline and with the incidence during follow-up of myocardial infarction, cardiovascular death and all-cause mortality in subjects with type 1 diabetes from GENESIS and GENEDIAB studies. The same allele was also inversely associated with the incidence of myocardial infarction and all-cause mortality during follow-up in the DIABHYCAR cohort of type 2 diabetic subjects. The protective T-allele of rs2284659 was associated with higher plasma EC-SOD and lower plasma AOPP concentrations in GENEDIAB.

AOPP concentration reflects the oxidation of plasma proteins, especially albumin, and is a reliable marker of oxidant-mediated protein damage [16]. Isoprostanes are a biologically active product of arachidonic acid metabolism formed as the result of oxygenation of polyunsaturated fatty acids. Circulating isoprostane concentration reflects lipid peroxidation associated with oxidative stress [11]. We observed higher plasma AOPP and isoprostane concentrations at baseline in type 1 diabetic subjects who died during the follow-up than in subjects who were alive at the end of the study. Isoprostane concentration at baseline was also higher in incident case of myocardial infarction. Previous studies have shown plasma AOPP and isoprostane to be an independent risk factors for coronary artery disease [10,11,19,20].

### SOD3 gene and risk of cardiovascular morbidity and mortality

Data from the literature on the association of the genes encoding the SOD enzymes with cardiovascular complications are scare. We have previously observed in the DIABHYCAR cohort associations of *SOD1* variants with cardiovascular mortality [21]. Mollsten and coworkers observed an association of a missense variant of *SOD2* with increased cardiovascular risk in a Danish cohort of type 1 diabetic patients [22]. Regarding *SOD3*, investigations dealt mostly with the rare functional variant rs1799895 (Arg213Gly) in the heparin-binding domain of EC-SOD. The Gly allele is associated with decreased EC-SOD affinity for heparin, decreased tissue binding, and a 6-to-10-fold increase in circulating EC-SOD concentration [17,23,24]. Despite higher circulating levels of the enzyme, the 213Gly isoform was shown to present only minimal anti-oxidant effects in the vascular wall [24], the loss of function being attributed to decreased tissue binding. Associations of the Gly allele with cardiovascular risk factors, morbidity or mortality were reported in a few studies [25,26].

Genetic analysis of rs1799895 in our study was inconclusive due to the very low frequency of the Gly allele in our cohorts. It is therefore unlikely that the associations we have observed with the promoter rs2284659 could be explained by a rs1799895 Gly allele effect. Moreover, linkage disequilibrium between the two variants was not strong (D’ = 0.687 and r^2^ = 0.009). The genetic mechanism behind the allelic associations that we have observed remains unclear, as rs2284659 does not seem to modify known transcription factor binding sites. However, it is noteworthy that rs2284659 is in complete linkage disequilibrium with two other SNPs in the promoter of *SOD3* (rs2159757 and rs13435617) that might affect transcription. The region surrounding and including rs2159757 matches the binding site for the PBX (pre-B-cell leukemia) family of transcription factors, and that surrounding and including rs13435617 matches the binding sites for HLF (hepatic leukemia factor) and E4BP4 (adenovirus E4 promoter-binding protein) transcription factors (http://consite.genereg.net). Interestingly, PBX transcription factors play a significant role in the morphogenesis, patterning and formation of the ventricular outflow tract (OFT). Mice lacking *PBX1* gene have a range of OFT malformations, including failure of cardiac septation [27]. A missense variant of *PBX3* (p.A136V) predicted to be deleterious for transcriptional function was four times more frequent in patients with congenital heart defects as compared to controls [28]. E4BP4 is implicated in the regulation of mammalian circadian oscillatory mechanism, anti-inflammatory response and cell survival. E4BP4 is expressed in the human heart and was shown to inhibit apoptotic proteins and to be overexpressed in cardiomyocytes following myocardial infarction [29]. The implication of these transcription factors on *SOD3* expression needs to be evaluated.

### Oxidative stress and vascular complications of diabetes

Oxidative stress is involved in the pathogenesis of atherosclerosis [30]. Increased production of reactive oxygen species (ROS) impairs endothelial function and endothelium-dependent vasodilation in humans, notably by inactivation of NO [31,32]. Oxidative stress also induces cell proliferation, hypertrophy, cardiac remodeling, apoptosis and inflammation in endothelial and smooth cells of the vascular wall and in myocardial cells [31,33,34]. Moreover, oxidative stress is associated with the metabolic syndrome and with many risk factors for atherosclerosis such as hypertension, dyslipidemia, obesity and diabetes [35]. Biomarkers of oxidative stress predict the risk of mortality in patients with heart disease [36]. Oxidative stress was shown to play a role in the premature vascular morbidity and mortality in people with diabetes [37]. Studies in animal models corroborate the implication of oxidative stress in cardiovascular morbidity and mortality related to diabetes [38,39].

Antioxidant enzymes such as the SODs scavenge ROS and inhibit NO degradation in the vascular wall [33]. The vascular wall contains large amounts of EC-SOD, which is produced and secreted to the extracellular space by smooth muscle cells [7,40]. The expression of *SOD3* was shown to be significantly reduced, and endothelium-bound EC-SOD activity to be correlated to flow-dependent endothelium-mediated dilation in subjects with coronary artery disease, suggesting that reduced EC-SOD activity might contribute to endothelial dysfunction in these patients [8]. Overexpression of *SOD3* in human vascular endothelial cells *in vitro* was shown to decrease endothelial-cell-derived superoxide production and LDL oxidation [9]. The implication of EC-SOD in cardiovascular protection is supported by studies in animal models. The overexpression of *SOD3* in transgenic mice was associated with protection of myocardial function after global ischemia/reperfusion [41]. Increased EC-SOD levels by adenovirus-mediated transfection of human *SOD3* cDNA was associated with protection against myocardial stunning and with decreased reperfusion infarct size in a rabbit model of ischemia/reperfusion injury [42]. Atherosclerosis was not increased in *SOD3* knockout mice given an atherogenic diet [43]. However, in an experimental model of focal cerebral ischemia, total infarct volume was 81% greater and hemiparesis more severe in *SOD3* knockout mice as compared to wild-type animals [44], while mice overexpressing *SOD3* had increased resistance to ischemia [45].

### Strengths and limitations

The main strengths of our study are the assessment of clinical phenotypes (myocardial infarction, cardiovascular and all-cause mortality) in three prospective cohorts of type 1 and type 2 diabetes and the genotyping of tagSNPs covering the haplotype block containing *SOD3*. We also investigated associations of the clinical phenotypes and *SOD3* genotypes with three markers of redox status. Overproduction of ROS and oxidative stress induced by chronic hyperglycemia are features of type 1 and type 2 diabetes [5]. Nevertheless, despite the observation in our study of similar genetic associations in both types of diabetes, we acknowledge that proper replication studies are needed in type 1 and type 2 diabetes cohorts. Our study has limitations, notably in issues related to the measurement of the redox biomarkers. AOPP and isoprostane were assayed in plasma-EDTA samples collected at baseline and kept frozen for ~20 years, and only in a subset of participants. These issues might have affected, at least in part, comparisons of plasma AOPP and isoprostane concentrations between groups. Moreover, measurements of redox biomarkers were performed only in GENEDIAB participants for whom plasma samples were available, and thus were not replicated in the present investigation. Another limitation of the study is the relatively small sample size of our type 1 diabetes cohorts and the small number of new cases of myocardial infarction during follow-up. The statistical power was adequate to detect SNP effects with HR ≥1.5, but might have been insufficient to detect effects of smaller magnitude.

## Conclusions

We have observed associations of rs2284659 in the promoter of the *SOD3* gene with myocardial infarction and with cardiovascular and all-cause mortality in subjects with type 1 or type 2 diabetes. The protective allele was associated with increased EC-SOD and decreased AOPP plasma concentrations, and thus with a more favorable plasma redox status. Our results suggest that EC-SOD plays a role in the mechanisms of vascular protection against oxidative stress in diabetic patients. Further studies are required to identify the functional variants and the molecular mechanisms beyond these allelic associations.

## Additional file

Additional file 1:
**Supplemental Tables.**

## Abbreviations

ACE: Angiotensin converting enzyme
ANCOVA: Analysis of covariance
ANOVA: Analysis of variance
AOPP: Advanced oxidation protein products
BMI: Body mass index
CI: Confidence intervals
eGFR: Estimated glomerular filtration rate
EC-SOD: Extracellular superoxide dismutase
HR: Hazard ratio
MAF: Minor allele frequency
MDRD: “Modification of Diet in Renal Disease” formula
OR: Odds ratio
ROS: Reactive oxygen species
SNP: Single nucleotide polymorphism
SOD: Superoxide dismutase
UAE: Urinary albumin excretion
