# Supplementary material for: Plasma extracellular superoxide dismutase concentration, allelic variations in the SOD3 gene and risk of myocardial infarction and all-cause mortality in people with type 1 and type 2 diabetes
Source: Cardiovasc Diabetol. 2015 Jan 15;14:845. doi: 10.1186/s12933-014-0163-2 (PMC4324771; doi:10.1186/s12933-014-0163-2)
Supplement: Additional file 1: — Supplemental Tables. [file 12933_2014_163_MOESM1_ESM.doc]

**Additional file 1: Table S1. GENEDIAB and GENESIS cohorts: Characteristics of participants at baseline**

|  | GENEDIAB | GENESIS |
| --- | --- | --- |
| N | 469 | 603 |
| Male sex (%) | 55.7 | 52.1 |
| Age (years) | 44.6 ± 12.4 | 42.0 ± 10.7 |
| Age at diabetes onset (years) | 15.9 ± 8.9 | 15.2 ± 8.6 |
| Duration of diabetes (years) | 28.5 ± 9.8 | 26.7 ± 9.0 |
| Body mass index (kg/m^2^) | 23.7 ± 3.2 | 24.3 ± 3.6 |
| HbA_1c_ (%) and (mmol/mol) | 8.6 ± 1.8 (70 ± 20) | 8.5 ± 1.4 (69 ± 15) |
| Systolic blood pressure (mmHg) | 138 ± 19 | 132 ± 20 |
| Diastolic blood pressure (mmHg) | 79 ± 12 | 75 ± 10 |
| Plasma creatinine (µmol/l) | 125 ± 115 | 114 ± 123 |
| eGFR (ml/min) | 71 ± 30 | 88 ± 52 |
| Urinary albumin excretion (mg/l) | 30 (401) | 21 (186) |
| Tobacco smoking (%) | 47.0 | 41.1 |
| Arterial hypertension (%) | 66.5 | 60.2 |
| Lipid lowering therapy (%) | 8.1 | 8.6 |
| Diabetic nephropathy stages (%) | 33/22/23/22 | 50/20/16/14 |
| Diabetic retinopathy stages (%) | 0/0/20/80 | 0/41/16/43 |
| Previous Myocardial Infarction (%) | 7.0 | 4.9 |

Results expressed as mean ± SD, except urinary albumin excretion (UAE) expressed as median and interquartile range. Statistics of quantitative parameters are ANOVA performed with log-transformed data, or Wilcoxon test (UAE). eGFR: estimated glomerular filtration. Diabetic nephropathy stages: absence, incipient, established, and advanced nephropathy. Diabetic retinopathy stages: absence, non-proliferative, pre-proliferative, proliferative retinopathy. p<0.05 is significant

**Additional file 1: Table S2. GENEDIAB/GENESIS pooled study:** Genotype frequency by the prevalence of previous myocardial infarction at baseline

| SNPs |  | Myocardial infarction at baseline | | | |
| --- | --- | --- | --- | --- | --- |
|  |  | No | Yes | OR (95% C.I.) | p |
| rs2284659 |  |  |  |  |  |
| GG |  | 0.402 (362) | 0.509 (27) | 0.48 (0.29 – 0.78) | 0.004* |
| GT |  | 0.447 (403) | 0.434 (23) |  |  |
| TT |  | 0.151 (138) | 0.057 (3) |  |  |
| MAF |  | 0.375 | 0.274 |  |  |
| rs1799895 |  |  |  |  |  |
| CC |  | 0.980 (877) | 0.982 (54) | - | ** |
| CG |  | 0.020 (18) | 0.018 (1) |  |  |
| GG |  | 0 | 0 (0) |  |  |
| MAF |  | 0.010 | 0.009 |  |  |
| rs2695234 |  |  |  |  |  |
| GG |  | 0.864 (784) | 0.902 (46) | 1.58 (0.66 – 3.30) | 0.26 |
| GA |  | 0.129 (117) | 0.039 (2) |  |  |
| AA |  | 0.007 (6) | 0.059 (3) |  |  |
| MAF |  | 0.071 | 0.078 |  |  |

| rs17552548 |  |  |  |  |  |
| --- | --- | --- | --- | --- | --- |
| AA |  | 0.905 (800) | 0.920 (46) | 0.99 (0.28 – 2.65) | 0.99 |
| GA |  | 0.093 (82) | 0.080 (4) |  |  |
| GG |  | 0.002 (2) | 0 (0) |  |  |
| MAF |  | 0.049 | 0.040 |  |  |
| rs758946 |  |  |  |  |  |
| TT |  | 0.861 (771) | 0.936 (44) | 0.53 (0.08 – 1.78) | 0.39 |
| TC |  | 0.131 (117) | 0.064 (3) |  |  |
| CC |  | 0.008 (7) | 0 (0) |  |  |
| MAF |  | 0.074 | 0.032 |  |  |
| rs2270224 |  |  |  |  |  |
| GG |  | 0.254 (220) | 0.204 (10) | 1.02 (0.62 – 1.69) | 0.85 |
| GA |  | 0.507 (443) | 0.612 (30) |  |  |
| AA |  | 0.239 (208) | 0.184 (9) |  |  |
| MAF |  | 0.492 | 0.490 |  |  |

Genotype data expressed as frequency and (number of cases). SNPs are sorted in 5' to 3' order. Odds ratio for the minor allele in a dominant or codominant* model, adjusted for sex, age, duration of diabetes, use of antihypertensive and lipid lowering medications, and for cohort membership. p≤0.01 is significant. MAF: minor allele frequency. **Statistics not computed due to low MAF (<0.02).

**Additional file 1: Table S3.** **GENEDIAB cohort: covariates associated with plasma EC-SOD levels at baseline in a stepwise regression analysis**

|  | Cumulated R^2^ | ß Coefficient | p |
| --- | --- | --- | --- |
| eGFR | 0.130 | -0.25 | <0.0001 |
| Sex (male) | 0.155 | -0.08 | 0.0002 |
| Systolic blood pressure | 0.179 | 0.65 | 0.0006 |
| BMI | 0.193 | -0.42 | 0.01 |
| Total cholesterol | 0.200 | 0.15 | 0.07 |
| Duration of diabetes | 0.203 | 0.003 | 0.19 |
| Age | 0.204 | 0.04 | 0.74 |
| Diastolic blood pressure | 0.204 | 0.07 | 0.74 |
| HbA1c | 0.204 | -0.02 | 0.85 |
| Urinary albumin excretion | 0.204 | 0.0001 | 0.92 |

The cumulated R^2^ expresses the percentage of the variance of the dependent variable (plasma EC-SOD levels) explained by the stepwise inclusion of covariates in the model (1=100%). Statistical analyses test if the covariate effect (ß Coefficient) is different from zero. p≤0.05 was considered significant. Analyses performed with log-transformed data.

**Additional file 1: Table S4. DIABHYCAR cohort: genotype frequency of *SOD3* variants by the incidence of clinical outcomes during follow-up**

|  |  | Myocardial Infarction during follow-up | | | |  | All-cause mortality | | | |
| --- | --- | --- | --- | --- | --- | --- | --- | --- | --- | --- |
|  |  | No | Yes | HR (95% C.I.) | p |  | No | Yes | HR (95% C.I.) | p |
| rs2284659 |  |  |  |  |  |  |  |  |  |  |
| GG |  | 0.395 (1148) | 0.489 (43) | 0.75 (0.59–0.94) | 0.01 |  | 0.390 (997) | 0.443 (194) | 0.87 (0.79–0.97) | 0.008 |
| GT |  | 0.459 (1332) | 0.398 (35) |  |  |  | 0.464 (1184) | 0.418 (183) |  |  |
| TT |  | 0.146 (424) | 0.113 (10) |  |  |  | 0.146 (373) | 0.139 (61) |  |  |
| MAF |  | 0.375 | 0.313 |  |  |  | 0.378 | 0.348 |  |  |
| rs1799895 |  |  |  |  |  |  |  |  |  |  |
| CC |  | 0.977 (2884) | 0.967 (88) | - | * |  | 0.976 (2540) | 0.982 (432) | - | * |
| CG |  | 0.022 (66) | 0.033 (3) |  |  |  | 0.023 (61) | 0.018 (8) |  |  |
| GG |  | 0.001 (2) | 0 (0) |  |  |  | 0.001 (2) | 0 (0) |  |  |
| MAF |  | 0.012 | 0.016 |  |  |  | 0.012 | 0.009 |  |  |
| rs2695234 |  |  |  |  |  |  |  |  |  |  |
| GG |  | 0.840 (2453) | 0.852 (75) | - | ** |  | 0.841 (2164) | 0.841 (364) | - | ** |
| GA |  | 0.148 (432) | 0.136 (12) |  |  |  | 0.147 (379) | 0.150 (65) |  |  |
| AA |  | 0.012 (34) | 0.012 (1) |  |  |  | 0.012 (31) | 0.009 (4) |  |  |
| MAF |  | 0.086 | 0.080 |  |  |  | 0.086 | 0.084 |  |  |

| rs17552548 |  |  |  |  |  |  |  |  |  |  |
| --- | --- | --- | --- | --- | --- | --- | --- | --- | --- | --- |
| AA |  | 0.892 (2640) | 0.946 (88) | 0.59 (0.29–0.98) | 0.04 |  | 0.892 (2325) | 0.904 (403) | 0.94 (0.79–1.09) | 0.42 |
| GA |  | 0.104 (308) | 0.043 (4) |  |  |  | 0.104 (272) | 0.090 (40) |  |  |
| GG |  | 0.004 (11) | 0.011 (1) |  |  |  | 0.004 (9) | 0.006 (3) |  |  |
| MAF |  | 0.056 | 0.032 |  |  |  | 0.056 | 0.052 |  |  |
| rs758946 |  |  |  |  |  |  |  |  |  |  |
| TT |  | 0.841 (2477) | 0.902 (83) | 0.73 (0.45–1.07) | 0.11 |  | 0.838 (2176) | 0.871 (384) | 0.89 (0.76–1.03) | 0.11 |
| TC |  | 0.155 (456) | 0.098 (9) |  |  |  | 0.158 (410) | 0.125 (55) |  |  |
| CC |  | 0.004 (13) | 0 (0) |  |  |  | 0.004 (11) | 0.004 (2) |  |  |
| MAF |  | 0.082 | 0.049 |  |  |  | 0.083 | 0.067 |  |  |
| rs2270224 |  |  |  |  |  |  |  |  |  |  |
| GG |  | 0.257 (741) | 0.231 (21) | - | ** |  | 0.260 (659) | 0.240 (103) | - | ** |
| GA |  | 0.474 (1364) | 0.450 (41) |  |  |  | 0.470 (1193) | 0.493 (212) |  |  |
| AA |  | 0.269 (773) | 0.319 (29) |  |  |  | 0.270 (687) | 0.267 (115) |  |  |
| MAF |  | 0.506 | 0.544 |  |  |  | 0.506 | 0.514 |  |  |

Genotype data expressed as frequency and (number of cases). SNPs are sorted in 5' to 3' order. Hazards ratio (HR) for the minor allele in a dominant model, determined by Cox proportional hazards survival regressive model, adjusted for sex, age, duration of diabetes, BMI, blood pressure, UAE, eGFR, hypertension and antiplatelet drugs and study treatment. p≤0.01 is significant. MAF, minor allele frequency. Statistics not computed due to low MAF (<0.02)* or absence of Hardy-Weinberg equilibrium**.
